# Medical-grade footwear: the impact of fit and comfort

**DOI:** 10.1186/s13047-016-0184-z

**Published:** 2017-01-05

**Authors:** Bessie Hurst, Helen Branthwaite, Andrew Greenhalgh, Nachiappan Chockalingam

**Affiliations:** Faculty of Health Sciences Staffordshire University, Leek Road, Stoke on Trent, ST4 2DF UK

**Keywords:** Comfort, Footwear, Prescription footwear, Pressure measurement, Footwear fit

## Abstract

**Background:**

Pressure-related skin lesions on the digits are a significant cause of discomfort. Most foot pain related to ill-fitting shoes occurs in the forefoot and digital areas. Pain has been associated with poor shoe fit, reduced toe box volume, as well as contour and shape of the shoe Off-the-shelf medical-grade footwear is designed as an intervention for chronic lesions on the digits. These shoes are designed with a flexible neoprene fabric upper that is thought to reduce pressure on the forefoot and reduce discomfort associated with ill-fitting shoes. The aim of this study was to investigate the effect of an off-the-shelf, medical-grade shoe on dorsal digital pressure and perceived comfort when compared to participant’s own preferred shoe.

**Methods:**

Thirty participants (18 females, 12 males) scored their perceived comfort whilst wearing each footwear style using a visual analog comfort scale. Dorsal digital and interdigital pressures were measured in using the WalkinSense® in-shoe pressure system. Sensors were placed on predetermined anatomical landmarks on the digits. Participants were randomly assigned the test shoe and their own shoe. Once wearing the shoe, the participants walked across a 6 m walkway and pressure data from each sensor was collected and processed to obtain peak pressure, time to peak pressure and contact time.

**Results:**

Participants scored the test shoe with higher comfort points than their own footwear. Overall peak pressure, pressure time integral and contact time decreased, whilst the time taken to reach peak pressure increased across all anatomical landmarks whilst wearing the test shoe. Statistically significant changes were observed for all of the measured variables relating to pressure on the medial border of the first metatarsophalangeal joint.

**Conclusion:**

The test shoe provided greater comfort and reduced the amount of pressure on the forefoot. The medical-grade footwear therefore, is a viable alternative to custom made prescription footwear and is more suitable than a regular everyday shoe when treating digital lesions associated with pressure.

## Background

The forefoot has been highlighted as the most frequent area of pain related to footwear [1]. Forefoot pain is commonly associated with wearing ill-fitting footwear [2, 3], causing pressure over bony prominences on the dorsum of the lesser toes, the medial aspect of the first metatarsal head or the lateral aspect of the fifth metatarsal [4]. In the long term, it is thought that the toes can adapt to footwear restrictions by extension of the metatarsophalangeal joints and flexion of the proximal interphalangeal joints [5]. Additionally, it has been shown that the forefoot is stiffer in habitually shod individuals and this loss of mobility may lead to greater incidences of forefoot pathology and deformity [6]. Digital deformities are subject to hyperkeratotic lesions, clavi, or ulcerations specifically on the interphalangeal joints which are subjected to frequent friction from adjacent toes [7]. A high proportion of corns are located on the dorsum of the 2^nd^, 3^rd^ and 4^th^ toes which often assume an elevated position as the first and fifth toes are forced to adapt to the confined area of the toe box of the shoe [8].

Recent studies conclude that wearing a shoe with a reduced toe box volume and shape may have poor foot health outcomes. It is suggested that this is caused by constriction of the toes which are associated with foot deformities including the development of joint pathologies and forefoot lesions [1, 9]. It is thought that shoes which do not have the capacity to accommodate the forefoot will alter the dynamics of the transverse metatarsal arch, restricting the metatarsal splay of the forefoot [6]. Poorly fitting footwear is thought to compress the digits and alter function, eventually leading to structural changes [10]. This compression can subsequently increase the pressure from the upper of the shoe on the toes and tissue breakdown/ulceration may occur [2, 11]. The design of the toe box related to depth and shape can impact the intensity of pressure, with a round styling on the medial border causing the least pressure and a pointed gradient to the lateral border improving pressure on the fifth digit [9]. However, this toe box shaping is not often seen in high street shoes contributing to incorrect fit of footwear.

Ill-fitting footwear is the primary cause of foot ulceration in patients who have systemic disease, such as diabetes, with 20% of presenting ulcers being due to shoes rubbing [12]. It has been shown that diabetic foot ulcerations are largely preventable when using custom off-loading footwear with ulcer recurrence rates found to decrease by 53% over a year follow up [13]. Re-ulceration rates were found to be 26% among therapeutic footwear group and 83% among those who wore their own footwear in ischemic and neuropathic ulcers, from a cohort of 386 ulcerations presented in clinic [14]. Off-loading pressure is therefore indispensable for stopping the potential progression of pre-ulcerative conditions toward lesions [9].

Although feet with significant deformities or delineated as high risk are recommended by NICE to be referred for bespoke footwear [15], there is a high dissatisfaction with prescription footwear to the point of not wearing them and they invariably become just another pair of “shoes in the cupboard” [16]. The lack of use of prescription footwear has been associated with the size, weight, design, comfort [17–19], lack of choice and styles of the shoes prescribed [20]. Although improved styling and newer materials used within the off-the-shelf medical-grade footwear (M-GF) has helped to increase compliance in wearing the shoe [21], there is still a lack of empirical data to support the use of these shoes for pressure reduction on the dorsum of the foot.

Therefore, this aims to explore the use of off-the-shelf medical-grade footwear as a pressure relieving intervention. Dorsal digital and interdigital forefoot pressure when wearing an off-the-shelf M-GF and the participants own footwear will be compared. Additionally, the comfort perception of both shoes will be assessed to evaluate if there is a difference between off the shelf M-GF and participants’ own footwear.

## Methods

Thirty participants (18 females, 12 males) from a convenience sample of routine podiatry patients with an average age of 71.4 years (M = 75.7, F = 68.5), height of 1.64 m (M = 1.7, F = 1.61), weight of 78.9 kg (M = 83.22, F = 74.34) and shoe size of 7 (M = 8.5, F = 6) were recruited from a UK private podiatry clinic. Ethical approval was sought and granted from Staffordshire University Ethics Committee and informed consent was provided by each participant. Participants were included in the study if they were male or female above 50 years of age, presenting with foot pain. Participants with musculoskeletal foot deformities including hallux valgus and lesser toe deformities were included in this research. Participants with a history of current ulceration, cognitive impairment, neurodegenerative disorder, peripheral neuropathy, impaired balance, amputation, wearing the intervention footwear or had use of foot orthoses three months previously, were excluded.

### Footwear characteristics

**Fig. 1:**
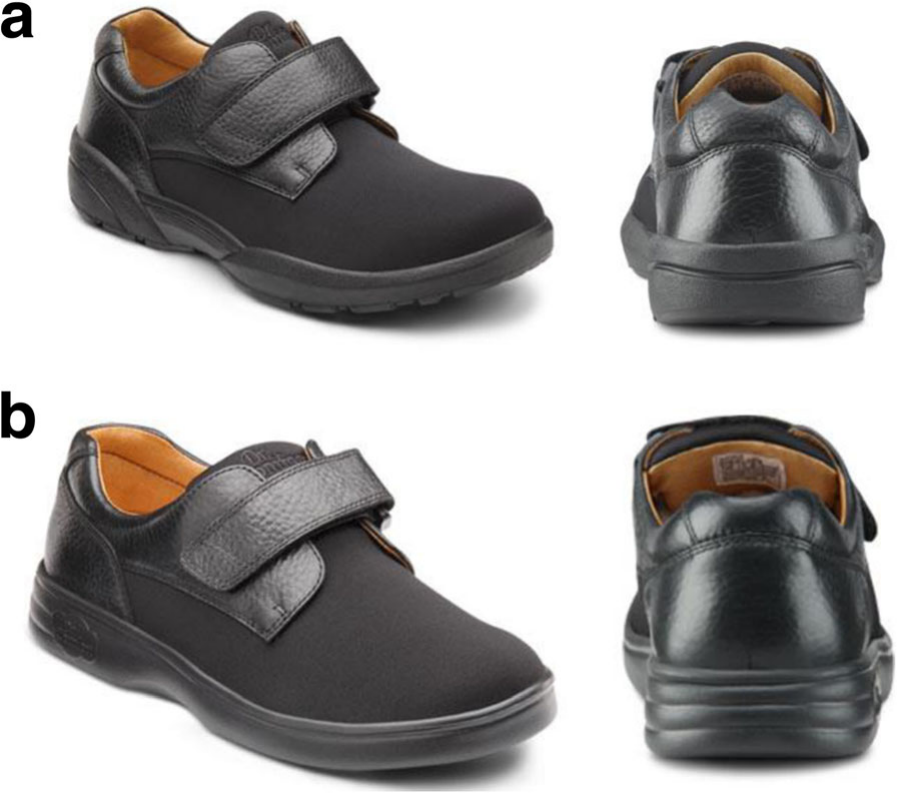
Dr Comfort Shoes (**a**) Brian for males and (**b**) Annie for females

The intervention footwear was selected from the Dr Comfort® (Vista, CA, USA https://www.drcomfort.com/), range of off-the-shelf medical-grade footwear. Two types of footwear were selected for this study; Brian (for men) and Annie (for women) both of the same styling, however the female shoe has reduced bulk to the sole unit (Fig. 1). The shoes are made with breathable and stretchable Lycra® (elastane) upper with Velcro ® fastening and seam free linings. The shoes are designed to accommodate most foot deformities including hammertoes and bunions and are offered in half sizes with three width fittings (medium, wide and extra wide) with extra width and depth in the toe box and forefoot. The footwear ranged in weight from 635 – 1100 g per pair including the removable insoles (a gel insole with a contoured heel cup, 7 mm thick under the forefoot and 15 mm thick under the heel with an additional flat 4 mm foam insert) both remained in the shoe during testing The footwear met the following set conditions for suitable footwear: a low heel, fastening, broad and deep toe box and a toe spring [18, 22].

The participants’ own footwear was the choice of shoe that was worn to attend the appointment, with no prior knowledge and instruction as to the suitable footwear criteria to prevent specific selection of shoe. Participants attended in a range of footwear including leather boots with fastenings and low heel profiles (maximum 2 cm), slip on leather court shoes with a heel height range of 3–5 cm, loafers and lace ups both with low heel profiles (maximum 2 cm). The participants own footwear was all purchased from a variety of high street outlets and had no defined medical features.

### Data collection

To ensure correct shoe size fitting, a footwear sizing measurement for each participant was taken using a Brannock device (The Brannock Device Company NY, USA) and MG-F was fitted accordingly. The participants own shoe was taken as the shoe worn to clinic and was not assessed for fit. The order of footwear testing was randomised by a card selection prior to data collection commencing.

### Comfort measure

A familiarisation period was allocated where participants were asked to walk along a 6 m walkway at a self-selected speed in the intervention shoe and the participants own shoe in the selected randomised order. Once completed for each shoe, participants were asked to rate initial comfort response by completing a 150 mm visual analogue comfort scale covering nine themes of footwear comfort [23]. The nine areas explored included; overall comfort, heel cushioning, forefoot cushioning, side to side support, arch height, heel fit, heel height, forefoot width and shoe length. Specific words that most clearly delineate extremes were anchored at the ends of the scale with the left labelled “not comfortable all” (0 comfort point) and the right end labelled, “most comfortable imaginable” (15 comfort points).

### In-shoe pressure system

**Fig. 2:**
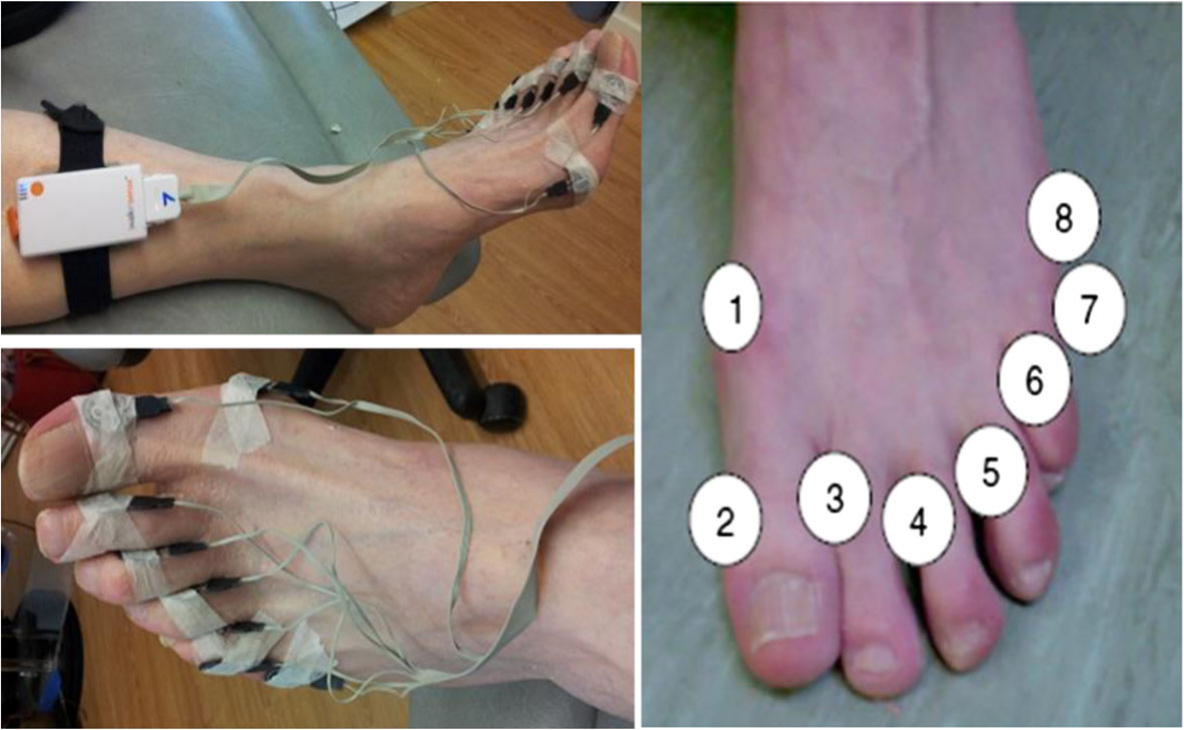
Walkinsense® sensor placement 1–8 on digital landmarks, as well as experimental set up for data capture. Micropore was loosely applied to secure the sensors

The WalkinSense® (Tomorrow Options SA, Porto, Portugal) system was used to gather digital toe pressure data whilst wearing the two footwear conditions in the same randomised order. This validated system [24] allows for individual sensors to be applied anywhere on the foot. Eight piezoresistive force, 100 Hz sensors were individually secured with Micropore™ tape (3 M, Bracknell, UK) to the following landmarks on the left foot [9] (Fig. 2).

(i) Medial border of the 1^st^ metatarsophalangeal joint
(ii) Medial border of the first interphalangeal joint
(iii) Interdigital (1/2, 2/3, 3/4, 4/5)
(iv) Proximal interphalangeal joint
(v) 5^th^ proximal interphalangeal joint
(vi) Lateral border of the 5^th^ metatarsal head

### Statistical analysis

#### Comfort scale

The comfort scale was measured by categorising nine footwear features including the overall comfort. Each characteristic was scored out of 150 mm and a total comfort score was calculated for both the M-GF and own footwear out of 1350 mm. The scores were averaged and statistically tested to find out which footwear provided the greatest level of comfort using Kruskal-Wallis test (SPSS v24 IBM USA) (*p* < 0.05).

#### In-shoe pressure system

Data was captured for a whole gait cycle with pressure analysed only during stance from the third and sixth footstep as these were identified as representing normal walking [25]. Pressure data was processed and averaged to obtain peak pressure, time to peak pressure and contact time. Each data set was assessed for normalcy and those test conditions meeting all parametric assumptions were statistically analysed using a paired samples *t*-test. Wilcoxon signed rank test was completed for data sets that did not meet parametric assumptions (SPSS v24 IBM USA) (*p* < 0.05).

## Results

The comfort scale rating for all footwear characteristics was consistently higher for the M-GF whilst the footwear length was considered the most comfortable characteristic. There were significant differences in all of the four pressure variables whilst wearing the M-GF. There was an overall decrease in peak pressure, pressure time integral and contact time, whilst the time taken to reach peak pressure increased with the M-GF. The medial border of the first metatarsophalangeal joint (sensor 1) consistently registered statistical significant difference (*p* < 0.05) across all four pressure variables.

### Comfort scale

The Kruskal-Wallis test revealed statistically significant difference in overall comfort perception (*p* < 0.05) (Table 1). Participants were consistent in scoring the length of both footwear with their highest comfort points and credited M-GF length as the most comfortable characteristic overall. This was closely followed by the ball of foot cushioning. The heel fit and side to side support registered with the least comfort points for M-GF and own footwear respectively. Cumulative the M-GF was more comfortable by 4.5 score points than the own footwear worn to clinic.

**Table 1:** Comfort perception of the nine themes in the M-GF and own shoe. Significant difference indicate by an asterisk (*)

		M-GF	Own Footwear	Sig 2-tailed (*p*-value)
Comfort Perception	Overall shoe comfort	11.57*	8.10	0.004
	Heel cushioning	11.32*	6.92	0.002
	Side to side support	10.98*	6.07	0.001
	Arch height	10.22	8.23	0.136
	Heel fit	10.05	6.33	0.123
	Ball of the foot width	10.27	7.80	0.18
	Heel width	11.35	9.30	0.124
	Ball of the foot cushioning	12.52*	7.32	0.007
	Length	13.10	9.57	0.043

### Mean peak pressure (PP)

**Fig. 3:**
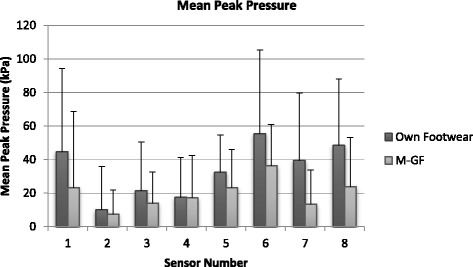
Mean peak pressure. Sensor 1 medial placement on the 1^st^ metatarsal moving around the digits to sensor 8 being placed on the 5^th^ metatarsal. Standard deviation indicated with error bars

Statistical tests showed a significant difference (Fig. 3) (*p* < 0.05) for sensors 1, 6, 7 and 8 and sensors 1 and 7 registering a high reduction of 79.35 and 66.83% of PP respectively with the M-GF. The eta squared statistics of 0.1 to 0.5 indicated a large effect size. M-GF shoes consistently reduced PP in all but sensor 3, which registered a marginal percentage increase (4.25%) in maximal load with the M-GF. The highest PP point was identified in sensor 6 (12.94 kPa, 24.07% increase) of participant’s own footwear, closely followed by sensor 8 (12.02 kPa, 36.90% increase).

### Mean time to peak pressure (TtPP)

**Fig. 4:**
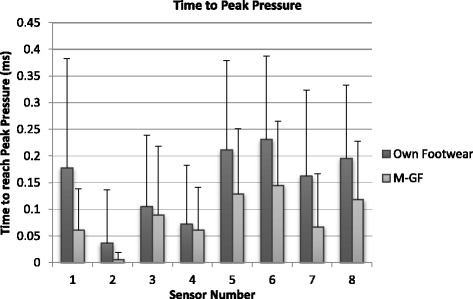
Mean time to peak pressure. Sensor 1 medial placement on the 1^st^ metatarsal moving around the digits to sensor 8 being placed on the 5^th^ metatarsal. Standard deviation indicated with error bars

The M-GF demonstrated earlier time to peak pressure in all 8 sensor (Fig. 4), although the difference in sensor 4 was marginal. Overall significant difference was recorded (*p* < 0.05). TtPP was significantly different in sensors 1, 7 and 8 (*p* < 0.05), with sensor 6 showing evidence of effect, but the result missed statistical significance. Overall, six of the eight anatomical regions demonstrated a large effect size (0.1 to 0.3).

### Mean contact time (CT)

**Fig. 5:**
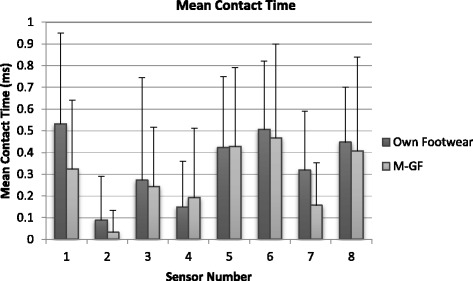
Mean contact time. Sensor 1 medial placement on the 1^st^ metatarsal moving around the digits to sensor 8 being placed on the 5^th^ metatarsal. Standard deviation indicated with error bars

Consistent decrease in ground CT was recorded with the M-GF in all 8 anatomical regions, but the differences were marginal in sensors 3, 4 and 5. Overall CT was statistically significantly (*p* < 0.05*)* (Fig. 5) whilst specific differences were observed in sensors 1, 6, 7, 8 (*p* < 0.05) with sensor 5 beginning to show statistical significance. Four regions demonstrated a large effect size (0.1 to 0.3).

## Discussion

Footwear choice is often made on comfort and activity [25]. Changing footwear habits can be difficult and perceptions of ill-fitting footwear contributing to pressure related toe problems are still not fully accepted. The results from this study indicate that the use of off-the-shelf medial grade footwear (M-GF) can significantly improve comfort and in addition reduce dorsal toe pressures in a clinical population.

M-GF were ranked most comfortable in all of the defined nine footwear characteristics and were in line with previous work which attest that the design, construction and properties of footwear are important factors in footwear comfort [26, 27]. The high comfort point for the ball of foot and heel cushioning are attributed to the M-GF’s lightweight cushioning polyeurethane sole, the removable flat 4 mm foam spacer and cushioning gel insole 7 mm thick under the forefoot, not always seen in routine footwear purchased from the high street. These structural features could be important to define clinical advice for comfortable footwear when ill-fitting footwear is chosen.

Footwear length was the most significant comfortable feature scored for participants from this podiatry practice, for each shoe condition indicating that the length of the shoe was well matched to foot size. It is thought that to obtain a good fit from a shoe that a distance of 1 cm is required at the end of the toe to allow for elongation during the gait cycle [22]. This has particular implications to lesser toe deformities which are associated with wearing shoes shorter than the foot [8]. Additionally wearing incorrect shoe length has been associated with foot, back pain and general biomechanical imbalance [1]. Although the participants own shoes were not measured, fitting of the M-GF shoe did include a foot sizing match. The differences in footwear comfort could have therefore been related to an improved fit from the M-GF shoe.

Footwear comfort perception is largely subjective [28], tactile, visual, auditory and olfactory sensations are involved in comfortable shoes selection [29, 30]. Despite initial concerns that the appearance and style of the intervention footwear may have resulted in negative perception of comfort and ultimately in low comfort scoring, participants appeared to exercise a high degree of objectivity in scoring, such that these factors did not adversely affects their scoring. This finding concurs with Williams et al. [18] that simply improving the appearance of shoes will improve patient compliance. Indeed it is possible that compliance may be improved by convincing patients to view therapeutic footwear as a prescription in a similar manner as the pharmacologic agents prescribed for medical needs.

Changing the perceptions of patients into believing that altering footwear style could improve pressure related foot problems is ongoing. It is clear from this study that the M-GF significantly altered all 3 pressure variables compared to the participants own footwear. However, it is not clear whether the participants’ own footwear was initially creating a pressure related foot problem that needed reducing. It is still unknown as to specific conditions for callus and other hyperkeratotic lesions to form. Yet, footwear shape has been identified as limiting foot function with the fifth toes often forced to adapt to the confined area of the toe box of footwear thus subjecting them to friction [8]. Similarly, toe box volume can compromise toe position and a wider forefoot has been correlated with ill-fitting footwear and pain [1].

The shape of the toe box is also important when considering pressure reduction [31]. The variance in shaping of the toe box allowed for a rounder wider toe box to be observed in the M-GF shoe over the participants own shoe. This altered shaping could explain why the difference in pressure, between the M-GF and participants own shoe, was reduced more at the sensors located on the medial and lateral borders of the foot rather than the sensors placed in between toes. There is some suggestion that the medial and lateral borders of the foot are the most frequent sites of foot pathology [32]. This may however be due to the unique mechanics of the joint mechanisms of the 1^st^ ray and the lateral column [33]. Yet, if dysfunction in foot mechanics exists in an individual the addition of ill-fitting footwear could exacerbate pressure related problems.

The limitations of this study include the potential for bias by not blinding the researcher or participant to footwear condition as this may have influenced subjective scoring. Similarly, comfort ratings of the shoes were recorded on the day of testing after only a brief familiarisation period therefore the results presented may not be considered an accurate indicator of the degree of comfort over longer periods of wear. The distribution of data between participants showed great variability leading to large standard deviations and an uneven spread across variables. Increasing the sample size could have rectified this error and should be considered for future work. Furthermore, this study could be a useful premise for the development of a larger scale structured clinical trial to explore the material properties and the construction of the shoe in relation to pathology. Future research would benefit from analysing the effects of the footwear on participant’s specific foot pathologies, anatomical variations, gait patterns, velocity and body weight.

Introducing M-GF in place of own footwear as an intervention for digital pressure related lesions will reduce the digital pressure and improve comfort. This type of footwear should be considered as part of a relevant treatment plan when discussing footwear choice with individuals.

## Conclusion

There is minimal research on the efficacy of appropriate non-bespoke pressure relieving footwear which clinicians can introduce to patients to purchase with confidence. Footwear catalogues such as Dr Comfort® are routinely given as part of a footwear advice with no structured research to support the use of the shoes. This study provides evidence to the efficacy of one style from the Dr Comfort® M-GF range which can be included in footwear advice protocols.
